# Prevalence, comorbidity and predictors of anxiety disorders in children and adolescents in rural north-eastern Uganda

**DOI:** 10.1186/1753-2000-7-21

**Published:** 2013-07-10

**Authors:** Catherine Abbo, Eugene Kinyanda, Ruth B Kizza, Jonathan Levin, Sheilla Ndyanabangi, Dan J Stein

**Affiliations:** 1 Department of Psychiatry, College of Health Sciences, Makerere University, P.O.BOX 7072, Kampala, Uganda; 2 Division of Child and Adolescent Psychiatry, Red Cross War Memorial Hospital and University of Cape Town, 7700 Rondebosch, Cape Town, South Africa; 3 MRC/UVRI Uganda Reseach Unit on AIDS, P.O.BOX 49, Entebbe, Uganda; 4 North Stockholm’s Psychiatric Clinic, Stockholm, Sweden; 5 Mental Health Division, Ministry of Health, Kampala, Uganda; 6 Department of Psychiatry and Mental Health, University of Cape Town, Cape Town, South Africa

**Keywords:** Children, Adolescents, Anxiety disorders, Comorbidity, Predictors, Uganda

## Abstract

**Background:**

Child and adolescent anxiety disorders are the most prevalent form of childhood psychopathology. Research on child and adolescent anxiety disorders has predominantly been done in westernized societies. There is a paucity of data on the prevalence, comorbidity, and predictors of anxiety disorders in children and adolescents in non-western societies including those in sub-Saharan Africa. This paper investigates the prevalence, comorbidity, and predictors of anxiety disorders in children and adolescents in north-eastern Uganda.

**Objective:**

To determine the prevalence of DSM-IV anxiety disorders, as well as comorbidity patterns and predictors in children and adolescents aged 3 to 19 years in north-eastern Uganda.

**Methods:**

Four districts (Lira, Tororo, Kaberamaido and Gulu) in rural north-eastern Uganda participated in this study. Using a multi-stage sampling procedure, a sample of 420 households with children aged 3–19 years from each district was enrolled into the study. The MINI International Neuropsychiatric Interview for children and adolescents (MINI KID) was used to assess for psychiatric disorders in 1587 of 1680 respondents.

**Results:**

The prevalence of anxiety disorders was 26.6%, with rates higher in females (29.7%) than in males (23.1%). The most common disorders in both males and females were specific phobia (15.8%), posttraumatic stress disorder (PTSD) (6.6%) and separation anxiety disorder (5.8%). Children below 5 years of age were significantly more likely to have separation anxiety disorder and specific phobias, while those aged between 14–19 were significantly more likely to have PTSD. Anxiety disorders were more prevalent among respondents with other psychiatric disorders; in respondents with two or more co-morbid psychiatric disorders the prevalence of anxiety disorders was 62.1%. Predictors of anxiety disorders were experience of war trauma (OR = 1.93, p < 0.001) and a higher score on the emotional symptom scale of the SDQ (OR = 2.58, p < 0.001). Significant socio-demograghic associations of anxiety disorders were found for female gender, guardian unemployment, living in permanent housing, living without parents, and having parents without education.

**Conclusion:**

The prevalence of anxiety disorders in children and adolescents in rural north-eastern Uganda is high, but consistent in terms of gender ratio and progression over time with a range of prior work in other contexts. Patterns of comorbidity and predictors of anxiety disorders in this setting are also broadly consistent with previous findings from western community studies. Both psychosocial stressors and exposure to war trauma are significant predictors of anxiety disorders.Prevention and treatment strategies need to be put in place to address the high prevalence rates of anxiety disorders in children and adolescents in Uganda.

## Background

Child and adolescent anxiety disorders are the most prevalent forms of childhood psychopathology, affecting about 10-20% of children and adolescents at some point in their lives [1-4]. Beesdo et al. (2009) reviewed studies that used instruments based on DSM III-TR and DSM IV. They reported a 6 month prevalence of anxiety disorders of 6.5 to 17.5% for DSM III-TR disorders and a 12 month prevalence of 6.9 to 17.7% for DSM IV disorders [5]. Their findings indicated that 3, 6 and 12 month prevalence of various anxiety disorders in children and adolescents are not considerably lower than lifetime prevalence [5]. Previous studies carried out in the west have reported that the most frequent psychiatric disorders in children and adolescents are separation anxiety disorder (with 3 months prevalence of around 4%), generalized anxiety disorder (GAD) (0.6% to 6.6%), specific phobias (0.2% to 10.9%), social phobia (0.6-7.0%) and panic disorder (0.0-1.2%) [3].

There is a particular paucity of data from sub-Saharan Africa. Previous work in Western Ethiopia, Ambo district has, however, reported a point prevalence for general childhood behavioral disorders of 17.7% with headache and nervousness as the most frequent symptoms [6]. In a study by Ensink and others in Khayelitsha, South Africa, a point prevalence of 21.7% was reported for PTSD in children aged 10–16 years [7]. In Acholiland, of which Gulu district is part, PTSD prevalence of 97% was reported in a 2004 study investigating former Ugandan child soldiers [8]. The same study found that even children who escaped from the rebel group- Lord’s Resistance Army a long time prior to the study continued to suffer from PTSD-like symptoms several years later [8]. In a comparative study of psychiatric disorders among war-abducted and non-abducted adolescents in Gulu district in Uganda, Okello and others reported that the rates of PTSD among the abducted group were more than twice that of the non-abducted group [9].

Different anxiety disorders have somewhat different age and gender distributions during childhood and adolescence. Separation anxiety and specific phobias are more common in preadolescent children, while panic disorder and social phobia are more common in adolescents [5]. Female children and adolescents have higher rates of anxiety disorder, with particularly high rates of specific phobia, PTSD and panic disorder (PD) [1,10].

Anxiety disorders in children and adolescents often co-exist with either another anxiety disorder or another psychiatric disorder. At least one third of children and adolescents with anxiety disorders meet criteria for two or more anxiety disorders [11]. Comorbidity of anxiety disorders and depression in children and adolescents, for example, is reported to range from 30% to 75%, and such comorbidity is associated with more severe anxiety symptoms [4,12,13] and greater suicidality [14].

There are a range of other predictors of anxiety disorders in children and adolescents. These include various indices of social disadvantage such as increased family size, overcrowding, low socioeconomic status, family disruptions, parental non-employment, father’s criminality and school disadvantage [15]. Again, most of the research on prevalence of and predictors for anxiety disorders in children and adolescents has been undertaken in the west, with only a few exceptions [15,16].

There is some evidence that anxiety disorders in non-western countries have the same comorbidity patterns as elsewhere, and may have similar predictors including age and gender [6]. However, further work is needed to confirm this preliminary impression. This paper aims to assess prevalence, comorbidity, and predictors of DSM-IV anxiety disorders in children and adolescents in north eastern Uganda. We focused on four rural districts, which are characterized by high poverty and low infrastructure. Two districts (Gulu, Lira) were also characterized by significant exposure to warfare.

## Methods

### Materials

The methods used in this study are described in detail elsewhere [13,14]. In summary, this study was conducted in the four districts of Lira, Tororo, Kaberamaido and Gulu in rural north-eastern Uganda. The study districts were selected from a list of eight districts where UNICEF was carrying out child directed medical and psychosocial interventions. In order to draw the sample of four study districts, the eight districts where UNICEF was undertaking child and adolescent directed activities were subdivided into two categories; those experiencing war conflict and those not experiencing such conflict at the time of the study. Two study districts were then randomly selected from each of these two categories. In the category of war affected districts Gulu and Lira were selected, while in the category of non-war affected districts Tororo and Kaberamaido were selected.

### Sampling procedure

Using Kish’s (1965) formula for cross-sectional studies and an average district population figure based on the Uganda Housing and Population Census of 2002, a 95% confidence interval, a precision of 4% and prevalence for emotional and behavioural problems of 15% [17,18], a sample size for each district of 420 households was estimated. To obtain this sample from each of the study districts, a multistage sampling procedure was used. During the first stage of sampling 2 sub-counties were randomly selected from a list of all sub-counties in each of the study districts. Where the district was war affected and had part of its population living in internally displaced persons camps (IDPs), the sub-counties in that district were initially divided into two groups, those that had IDPs and those that did not, then from each of these two groups a sub-county was randomly selected.

At the next stage, all the parishes in the selected sub-counties were listed and a parish randomly selected. All households in the selected parish were then listed and households with children and adolescents aged 3–19 years were consecutively enrolled into the study until the sample of 210 households per sub-county was attained. If the sample size of 210 households with children aged 3–19 years could not all be obtained from a single parish, a second parish was then randomly selected from the list of parishes in that study and sub-county and households were recruited from there until the required sample was obtained.Where a selected household had more than one child or adolescent who was less than 19 years of age, only one study respondent was selected by simple random sampling.

### Measures

A generic survey instrument was compiled and translated into the main dialects spoken in the selected sub-counties. To ensure semantic equivalence between English and the local dialects, a process of forward and back translation was undertaken. For each of the 4 main dialects spoken in the study sub-counties, two teams of mental health professionals were constituted. The first team translated these two psychological assessment tools into the local dialect and the second team (which was blind to the initial English version) translated the local dialect version into English. A consensus meeting with the two teams was then held and any major differences in the two versions resolved by discussion.

The translated survey instrument was then administered by trained psychiatric nurses for each selected child or adolescent aged 3–19 years. The trained psychiatric nurses interviewed the children and adolescents themselves (for those who were 10 years or older and capable of responding verbally) or their mothers (for those who were aged less than 10 years or not capable of responding verbally).

The survey instrument contained the following sections:

i) Emotional and behavioural problems

The Strengths and Difficulties questionnaire (SDQ) [19], was used to assess emotional and behavioural problems. This is a 25- item questionnaire that can be administered to parents or teachers of 3–16 year olds or directly to 11–16 year olds to screen for psychological distress. It covers common areas of emotional and behavioural difficulties and has been validated in both western and developing country settings. The 25 items of the SDQ are divided into 5 subscales of 5 items each, which measure emotional symptoms, conduct problems, hyperactivity/inattention, peer relationship problems and prosocial behaviour, and which taken together comparise a total difficulties score [19].

The SDQ is scored using a Likert scale with the following scores; 0 = not true, 1 = somewhat true and 2 = certainly true. On the basis of an ROC analysis restricted to children and adolescents aged 3–16 using having ‘at least one DSM-IV psychiatric diagnosis’ as a ‘gold standard’, a score of at least 16 was chosen as indicative of psychological distress in children and adolescents. This score ensured a sensitivity of above 60% while keeping adequate specificity [13,14].

ii) DSM IV psychiatric disorders

The MINI International Neuropsychiatric Interview for children and adolescents (M.I.N.I.-KID) [20,21], which embodies DSM-IV-TR criteria for various psychiatric disorders in children and adolescents was used to make specific psychiatric diagnoses. The MINI-KID screens for 23 axis 1 diagnoses. For most modules of the MINI-KID, two to four screening questions are used at the beginning of each module [20,21]. Further diagnostic questions are asked if the response to screening questions is positive [21]. For each diagnostic category, DSM-IV-TR has a specific number of symptoms, often a duration of disturbance and a distress or impairment criterion [20,21].

To construct syndrome categories for analysis, these psychiatric disorders were grouped as follows: depressive disorder syndromes (major depressive episode, dysthymia); psychotic disorder syndromes (manic episode, psychotic disorder); anxiety disorder syndromes (panic disorder, agoraphobia, separation anxiety disorder, social phobia, specific phobia, obsessive-compulsive disorder, PTSD, generalized anxiety disorder, adjustment disorder); alcohol and substance abuse disorder syndromes (alcohol abuse and dependency, non-alcohol psychoactive substance use disorder); neurodevelopmental disorders (conduct disorder, oppositional deficit disorder, pervasive development disorder, attention deficit hyperactivity disorder (ADHD) combined disorder, ADHD hyperactive/ impulsive disorder, and ADHD inattentive disorder); eating disorders (anorexia nervosa, bulimia nervosa) and tic disorders (motor tic disorder, vocal tic disorder, Tourette’s disorder, transient tic disorder). Suicidality was defined as meeting any of the three criteria for past suicidality provided in the MINI International Neuropsychiatric Interview for children and adolescents: i) have you ever felt so bad that you wished you were dead ? ii) have you ever tried to hurt yourself? iii) have you ever tried to kill yourself? [21].

iii) Socio-demographic variables

A socio-demographic questionnaire included the following variables: a) the subject’s age, gender, tribe, resident district, highest level of education attained and history of exposure to war trauma ; b) previous history of mental illness (psychosis) and attendance at a mental health facility and c) current living arrangement (living with both parents, mother alone, father alone, friends, adopted parents, grandparents and other relatives), orphanhood status, number of siblings, parents’/ guardians’ employment status, family’s total income per month (in Uganda shillings), parents’ highest educational attainment, exposure to domestic violence in the home, nature of housing (permanent or hut and others) and family history of severe mental illness (psychosis).

Additional variables considered in this study included assessment for exposure to war trauma (by asking the question: ‘have you been involved in a situation of war trauma [lived in an IDP, witnessed the torture/ killing of someone, suffered physical or sexual violence as a results of war, been abducted or threatened with violence as a result of war]).

### Ethical approval

The study obtained Ethical Clearance from the Ministry of Health and the Uganda National Council of Science and Technology. Respondents 18 years and above were required to provide informed consent, while respondents below the age of 18 years were required to provide assent as well as the consent of a parent/guardian.

### Statistical analysis

The prevalence of anxiety disorders was estimated. In order to assess factors associated with anxiety disorders the approach of Victoria and others was followed [22]. Firstly the association of socio-demographic factors was investigated using a backward elimination regression model, choosing the candidate variables based on prior knowledge and plausibility, and using a liberal p-value (15%) to ensure that all variables with a possible confounding effect on the ultimate risk factors were included [23].The socio-demographic factors selected were then all included in a second stage model in which candidate predictors were added and removed using a backward elimination algorithm with a stricter 5% p-value. The results were checked by carrying out forward selection with all selected socio-demographic variables and the same candidate predictors. All analyses were carried out using Stata release 11.2 (StataCorp., College Station, Texas).

## Results

Psychiatric disorders and co-morbidities were assessed in 1587 (94.5%) of 1680 respondents. The main reason for not being able to assess non-respondents was repeated absence from the home.

### Prevalence

The overall prevalence of anxiety disorders in this study was 26.6%, which was higher in females (29.7%) than in males (23.1%). The prevalence of specific anxiety disorders is given in Tables 1 and 2. The most common anxiety disorder in both males and females was specific phobia (15.8% ) followed by PTSD (6.6%) and separation anxiety disorder (5.8%). The prevalence of every disorder was higher among females than among males. Younger children (aged below 5 years) were significantly more likely to have separation anxiety disorder (7.7%,) and specific phobias (20.3%), while those aged 14–19 were significantly more likely to have PTSD (12.8%).

**Table 1:** Prevalence of anxiety disorders in males and females

**Disorder**	**Males (n = 734)**	**Females (n = 853)**	**Total (n = 1587)**
Any anxiety disorder	172 (23.4%)	251 (29.4%)	423 (26.6%)
	(20.4% - 26.7%)	(26.4% - 32.6%)	(24.5% - 28.9%)
Panic disorder	15 (2.0%)	33 (3.9%)	48 (3.0%)
	(1.1% - 3.3%)	(2.7% - 5.4%)	(2.2% - 4.0%)
(b) Agoraphobia	25 (3.4%)	35 (4.1%)	60 (3.8%)
	(2.2% - 5.0%)	(2.9% - 5.7%)	(2.9% - 4.8%)
Separation anxiety disorder	40 (5.4%)	52 (6.1%)	92 (5.8%)
	(3.9% - 7.3%)	(4.6% - 7.9%)	(4.7% - 7.1%)
Social phobia (social anxiety disorder)	36 (4.9%)	47 (5.5%)	83 (5.2%)
	(3.5% - 6.7%)	(4.1% - 7.3%)	(4.2% - 6.4%)
Specific phobia	97 (13.2%)	153 (17.9%)	250 (15.8%)
	(10.8% - 15.9%)	(15.4% - 20.7%)	(14.0% - 17.6%)
Obsessive compulsive disorder	3 (0.41%)	6 (0.70%)	9 (0.57%)
	(0.08% - 1.19%)	(0.26% - 1.52%)	(0.26% - 1.07%)
Post-traumatic stress disorder	46 (6.3%)	59 (6.9%)	105 (6.6%)
	(4.6% - 8.3%)	(5.3% - 8.8%)	(5.4% - 8.0%)
Generalized anxiety disorder	4 (0.54%)	18 (2.1%)	22 (1.4%)
	(0.15% - 1.40%)	(1.3% - 3.3%)	(0.87% - 2.09%)
Adjustment disorder	3 (0.41%)	5 (0.59%)	8 (0.50%)
	(0.08% - 1.2%)	(0.19% - 1.4%)	(0.22% - 0.99%)

**Table 2:** Prevalence of anxiety disorders in different age groups

**Disorder**	**<=5 (n = 286)**	**6-9 (n = 416)**	**10-13 (n = 550)**	**14-19 (n = 335)**	**Total (n = 1587)**
Any anxiety disorder	72 (25.2%)	103 (24.8%)	148 (26.9%)	100 (29.9%)	423 (26.7%)
	(20.3%- 30.6%)	(20.7%-29.2%)	(23.2%-30.8%)	(25.0%35.1%)	(24.5%-28.9%)
Panic disorder	5 (1.7%)	13 (3.1%)	14 (2.5%)	16 (4.8%)	48 (3.0%)
	(0.6% - 4.0%)	(1.7% - 5.3%)	(1.4% - 4.2%)	(2.8% - 7.6%)	(2.2% - 4.0%)
(b) Agoraphobia	9 (3.1%)	20 (4.8%)	15 (2.7%)	16 (4.8%)	60 (3.8%)
	(1.4% - 5.9%)	(3.0% - 7.3%)	(1.5% - 4.5%)	(2.8% - 7.6%)	(2.9% - 4.8%)
Separation anxiety disorder	22 (7.7%)	30 (7.2%)	30 (5.5%)	10 (3.0%)	92 (5.8%)
	(4.9% - 11.4%)	(4.9% - 10.1%)	(3.7% - 7.7%)	(1.4% - 5.4%)	(4.7% - 7.1%)
Social phobia (social anxiety disorder)	13 (4.5%)	24 (5.8%)	29 (5.3%)	17 (5.1%)	83 (5.2%)
	(2.4% - 7.6%)	(3.7% - 8.5%)	(3.6% - 7.5%)	(3.0% - 8.0%)	(4.2% - 6.4%)
Specific phobia	58 (20.3%)	67 (16.1%)	81 (14.7%)	44 (13.1%)	250 (15.8%)
	(15.8%- 25.4%)	(12.7%-20.0%)	(11.9%-18.0%)	(9.7%-17.2%)	(14.0%-17.6%)
Obsessive compulsive disorder	0	4 (0.96%)	4 (0.73%)	1 (0.30%)	9 (0.57%)
	(0–1.3%)	(0.26% - 2.4%)	(0.20% - 1.9%)	(0.01%-1.7%)	(0.26%-1.07%)
Post-traumatic stress disorder	0	19 (4.6%)	43 (7.8%)	43 (12.8%)	105 (6.6%)
	(0 – 1.3%)	(2.8% - 7.0%)	(5.7% - 10.4%)	(9.4%-16.9%)	(5.4% - 8.0%)
Generalized Anxiety disorder	0	8 (1.9%)	9 (1.6%)	5 (1.5%)	22 (1.4%)
	(0 – 1.3%)	(0.8% - 3.8%)	(0.8% - 3.1%)	(0.5% - 3.4%)	(0.87%-2.09%)
Adjustment disorder	0	3 (0.7%)	3 (0.5%)	2 (0.6%)	8 (0.50%)
	(0 – 1.3%)	(0.1% - 2.1%)	(0.1% - 1.6%)	(0.1% - 2.1%)	(0.22%-0.99%)

### Association of socio-demographic variables with anxiety disorders

The association of anxiety disorders with socio-demographic factors is summarized in Table 3. The prevalence of anxiety disorders is lowest in Gulu and highest in Lira, the two of the districts that had IDP camps. Anxiety disorders are more common in participants who are older, have some secondary education, live with their father only or with grandparents, have 7 or more siblings, live in permanent housing, have parents with no formal education, or guardians who are unemployed and lowest amongst those whose parents had secondary or higher education.

**Table 3:** Sociodemographic factors and anxiety disorders: Bivariate associations

**Factor**	**Level**	**Total (n)**	**Anxiety disorders n(%)**
District	Gulu	403	22 (5.5%)
	Kaberamaido	399	128 (32.1%)
	Lira	372	147 (39.5%)
	Tororo	413	126 (30.5%)
Age in years (grouped)	≤ 5	286	72 (25.2%)
	6 – 9	416	103 (24.8%)
	10 – 13	550	148 (26.9%)
	14 – 19	335	100 (29.8%)
Gender	Male	734	172 (23.4%)
	Female	853	251 (29.4%)
Education	No formal education	409	115 (28.1%)
	Primary (1 – 7 years)	1120	286 (25.5%)
	Secondary (8+ years)	58	22 (37.9%)
Living arrangements	With both parents	945	222 (23.5%)
	Mother Only	336	84 (25.0%)
	Father Only	56	23 (41.1%)
	With grandparents	132	56 (42.4%)
	Other	118	38 (32.2%)
Nature of housing	Permanent	133	46 (34.6%)
	Semi-permanent	297	72 (24.2%)
	Hut	1105	282 (25.5%)
	Other	52	23 (44.2%)
Family income (UGX)	< 15,000	777	216 (27.8%)
	15,000 – 99,000	358	109 (30.4%)
	100,000 +	285	67 (23.5%)
Employment status of guardian	Professional	338	77 (22.8%)
	Casual	382	49 (12.8%)
	Housewife	216	63 (29.2%)
	Unemployed	410	153 (37.3%)
	Other	241	81 (33.6%)
Number of siblings	0 – 2	243	65 (26.8%)
	3 - 4	421	111 (26.4%)
	5 - 6	493	114 (23.1%)
	7+	430	133 (30.9%)
Parent’s education	None	400	124 (31.0%)
	Elementary (Primary)	841	233 (27.7%)
	Secondary	217	40 (18.4%)
	Higher	129	26 (20.2%)

### Association of psychiatric and psychosocial variables with anxiety disorderss

Table 4 shows the association of anxiety disorders with psychiatric and psycho-social variables. Anxiety disorders were more prevalent among respondents with other psychiatric disorders and for the 66 subjects (4.1%) who had two or more co-morbidities the prevalence of anxiety disorders was 62.1%. The prevalence of anxiety disorders was higher among subjects whose parents were not both alive, those with a history of serious mental illness, those with emotional and behavioural problems as measured by an SDQ score of 16 or higher, and those with abnormal or borderline scores on the emotional symptoms scale.

**Table 4:** Association of psycho-social factors with anxiety in children / adolescents

**Factor**	**Level**	**Total (n)**	**Anxiety syndromes n(%)**
Depression	No	1451	346 (23.8%)
	Yes	136	77 (56.6%)
Psychotic disorder syndromes	No	1563	410 (26.2%)
	Yes	24	13 (54.2%)
Suicidality	No	1502	368 (24.5%)
	Yes	85	55 (64.7%)
Alcohol and substance abuse	No	1565	416 (26.6%)
	Yes	22	7 (31.8%)
Motor disorder syndromes	No	1574	414 (26.3%)
	Yes	13	9 (69.2%)
Behavioral developmental disorder syndromes	No	1508	400 (26.5%)
	Yes	79	23 (29.1%)
Eating disorders	No	1576	412 (26.1%)
	Yes	11	11 (100%)
Number of DSM disorders	0	1320	305 (23.1%)
	1	201	77 (38.3%)
	2 or more	66	41 (62.1%)
Family history of mental illness	None	1175	316 (26.9%)
	First Degree relative	156	46 (29.5%)
	Other relative	256	61 (23.8%)
Experience of trauma	No	1023	270 (26.4%)
	Yes	564	153 (27.1%)
Parents alive	Yes	1069	260 (24.3%)
	No	518	163 (31.5%)
History of mental illness (attendance at facility)	No	1517	395 (26.0%)
	Yes	70	28 (40.0%)
Emotional and behavioral problems	Non-case (SDQ < 16)	918	175 (19.1%)
	Case (SDQ ≥ 16)	669	248 (37.1%)
Emotional symptoms scale	Normal	1045	198 (19.0%)
	Borderline	173	61 (35.3%)
	Abnormal	272	126 (46.3%)
	Missing	97	38 (39.2%)

### Multiple logistic regression model

The results of a multiple logistic regression model including the variables identified as potential socio-demographic determinants of anxiety is given in Table 5. Adjusting for district, gender, employment status of guardian, living arrangements, nature of housing, subjects’ education, parents’ education and number of siblings, the factors found to be significantly associated with anxiety disorders were experience of trauma (OR = 1.93, 95% p < 0.001), score on the emotional symptom scale (OR = 2.58, p < 0.001), and presence of DSM disorders (OR = 3.06, p = 0.001). Significant associations of anxiety disorders were found for female gender (OR = 1.38, p = 0.016), guardian unemployment (OR = 2.16, p < 0.001), living with father only (OR = 2.22, p = 0.005), living in permanent housing, and having parents without education(OR = 0.60,p = 0.049). At both model selection stages (choosing the socio-demographic factors and choosing the psycho-social factors) forward selection confirmed the factors chosen by backward elimination. There was no evidence of any significant interaction effects between sociodemographic and other variables.

**Table 5:** Results of fitting multiple logistic regression models for factors associated with anxiety in children / adolescents

**Factor**	**Level**	**Odds ratio (95% confidence interval)**	**Likelihood ratio Test P-value**
District	Gulu	1 (Reference Level)	<0.001
	Kaberamaido	13.7 (7.9 ; 23.6)	
	Lira	12.6 (7.4 ; 21.5)	
	Tororo	11.7 (6.7 ; 20.4)	
Gender	Male	1 (Reference Level)	0.016
	Female	1.38 (1.06 ; 1.79)	
Employment status of guardian	Professional	1 (Reference Level)	<0.001
	Casual	0.41 (0.25 ; 0.68)	
	Housewife	1.39 (0.83 ; 2.33)	
	Unemployed	2.16 (1.38 ; 3.36)	
	Other	1.43 (0.90 ; 2.28)	
Living arrangements	Both Parents	1 (Reference Level)	0.05
	Mother Only	0.99 (0.70 ; 1.41)	
	Father Only	2.22 (1.18 ; 4.19)	
	With Grandparents	1.55 (0.96 ; 2.50)	
	Other	1.34 (0.83 ; 2.18)	
Nature of housing	Permanent	1 (Reference Level)	<0.001
	Semi-permanent	0.37 (0.22 ; 0.63)	
	Hut	0.39 (0.23 ; 0.65)	
	Other	0.76 (0.35 ; 1.68)	
Parents education level	None	1 (Reference Level)	0.049
	Elementary	0.96 (0.70 ; 1.33)	
	Secondary	0.60 (0.37 ; 0.98)	
	Other	0.54 (0.29 ; 1.00)	
Number of siblings	0 - 2	1 (Reference Level)	0.098
	3 – 4	1.11 (0.73 ; 1.71)	
	5 – 6	0.84 (0.55 ; 1.29)	
	7+	1.28 (0.83 ; 1.97)	
Education	No formal education	1 (Reference Level)	0.090
		0.79 (0.58 ; 1.08)	
		1.40 (0.70 ; 2.79)	
Experience of trauma	No	1 (Reference Level)	<0.001
	Yes	1.93 (1.42 ; 2.62)	
Emotional symptom Scale	Normal	1 (Reference Level)	<0.001
	Borderline	2.00 (1.34 ; 2.99)	
	Abnormal	2.58 (1.82 ; 3.65)	
	Missing	2.19 (1.34 ; 3.59)	
Number of DSM disorders	None	1 (Reference Level)	0.001
	One	1.19 (0.82 ; 1.74)	
	Two or More	3.06 (1.67 ; 5.62)	

## Discussion

In this study, the overall prevalence of anxiety disorders was 26.6%, with rates higher in females (29.7%) than in males (23.1%). The most common disorders in both males and females were specific phobia (15.8%), PTSD (6.6%) and separation anxiety disorder (5.8%). Children below 5 were significantly more likely to have separation anxiety disorder and specific phobias , while those aged between 14–19 were significantly more likely to have PTSD. Anxiety disorders were more prevalent among respondents with other psychiatric disorders; in respondents with two or more co-morbid psychiatric disorders the prevalence of anxiety disorders was 62.1%.

Our finding of a 26.6% point prevalence of anxiety disorders is about two and half times higher than rates reported in community studies in western countries [3,24,25]. Contextual differences may be one explanation for the higher prevalence as compared to the studies done in the west. Several districts have been negatively affected by the presence of rebels [26]. Some regions have suffered cattle rustling from neigbouring karamojongs, drought and floods. Poverty levels in the rural areas of Uganda are 3 to 4 times higher than in urban areas [26], and the districts have limited infrastructure. These findings here are consistent with previous work noting an an association of chronic psychosocial stressors with onset of anxiety disorders in children [27], as well as with a literature on this relationship in adults [28].

Many of the earlier community studies used DSM-III and DSM-III-TR criteria, which differ from the DSM-IV criteria used in our study. Such methodological differences may also contribute to variation in prevalence estimates across different studies. Given the clinical criterion, comprising distress or functional impairment embodied in the DSM-IV diagnostic criteria, one would arguably expect more conservative prevalence estimates than obtained with earlier criteria. Some of the high prevalence rates, particularly in males, are therefore concerning; for example, high rates of panic disorder in early life in both genders is an usual finding that may reflect the high rates of psychosocial stressors faced by our respondents.

Other findings reported here are similar to those reported in previous community studies of anxiety disorders in children and adolescents. Thus, multiple studies confirm increased prevalence of anxiety disorders in females [5]. Similarly, many studies have previously shown that there is a specific developmental progression in the onset of anxiety disorders, with specific phobias and separation anxiety disorder having the earlier age of onset [5,29].

Comorbidity findings here also share a great deal in common with previous community studies on anxiety disorders in children and adolescents [30,31]. We found that in respondents with two or more co-morbid psychiatric disorders the prevalence of anxiety disorders was 62.1%; Similarly, previous work has consistently found that respondents with anxiety disorders have elevated comorbidity [31]. Furthermore, such comorbidity is associated with increased symptom severity as well as greater functional impairment and worse outcome [32,33].

Predictors of anxiety disorders included experiencing war trauma, female gender, guardian unemployment, living without parents, and parents without education. Such findings are consistent with a prior literature indicating multiple associations between anxiety disorders and psychosocial stressors [27,28,34]. This may point to the importance of “everyday” chronic stressors, in addition to exposure to war trauma per se, in the pathogenesis of anxiety disorders. Some of our findings, such as the association between anxiety disorders and living in a permanent home were a surprise prediction and deserve further study to determine replicability.

The results of this study should be considered in light of a number of limitations. First, the design of the study was cross-sectional and therefore any causal attributions are tentative at best. Further studies using prospective designs would help in examining cause and effect relationships. There is a growing database of work on the long-term negative effects of childhood and adolescent anxiety disorders. For example, in one prospective study, first graders (ages 5 and 6 years) who reported high levels of anxiety symptoms were at a significant risk of persistent anxiety symptoms and low achievement scores in reading and maths in fifth grade ( age 10 years) [35]. More recently, Grover et al. reported that early-onset anxious symptoms in African American children were associated with both concurrent and long-term academic, social, and psychological difficulties [36]. In the Ugandan setting, further work is needed to tease out the relationships between exposure to psychosocial stressors and to war trauma.

Second, although we paid careful attention to semantic equivalence in our translations, and although there seem to be many universal aspects of anxiety disorders symptoms, the possibility that the diagnostic instruments used here may not have captured culture-specific aspects of anxiety disorders in Uganda cannot be ruled out [37,38]. Finally, given that younger children may have difficulties in communicating information about internally experienced affective states, the use of an interview based on DSM-IV diagnostic criteria may be particularly problematic, despite the use of parental interviewees [39].

## Conclusions

In summary, the prevalence of anxiety disorders in children and adolescents in rural north-eastern Uganda is high, but consistent in terms of gender ratio and progression over time with a range of prior work in high, middle, and low income countries [40]. Patterns of comorbidity and predictors of anxiety disorders in this setting are also broadly consistent with previous findings from western community studies. It is notable that both chronic psychosocial stressors and exposure to war trauma are significant predictors of anxiety disorders.Both prevention and treatment strategies need to be put in place to address the high prevalence rates of anxiety disorders in children and adolescents in Uganda.

## Abbreviations

DSM III: Diagnostic and Statistical Manual, Third Edition; DSM III-TR: Diagnostic and Statistical Manual, Third Edition Text Revised; DSM IV: Diagnostic and Statistical Manual, fourth Edition; PTSD: Post Traumatic Stress Disorder; GAD: Generalised Anxiety Disorder; PD: Panic Disorder; UNICEF: United Nations Children’s Fund; APFP: African Paediatric Fellowship Programme.

## Competing interests

The authors declare no competing interests.

## Authors' contributions

CA, EK, RK, and SN were all involved in the conceptualization, proposal writing and supervision of data collection. JL analyzed data and wrote the methods and results section of this manuscript. CA drafted the rest of sections of the manuscript. DJS made critical revision of the manuscript for important intellectual content. All authors have read the whole manuscript and made their contributions. All authors read and approved the final manuscript.

## Authors' information

CA: Lecturer and a psychiatrist in the Department of Psychiatry, currently Senior Registrar, Division of Child and Adolescent Psychiatry,Red Cross War Memorial Hospital, University of Cape Town and a fellow, African Peadiatric Fellowship Programme.

EK: Consultant Psychiatrist, Research Manager MRC/UVRI Uganda Research Unit on AIDS

RK: Psychiatrist, North Stockholm’s Psychiatric Clinic, Stockholm, Sweden

JL: Senior Statistician, MRC/UVRI Uganda Research Unit on AIDS

SN: Pincipal Medical Officer, Coordinator, Mental Health Division, Ministry of Health, Uganda

DJS: Head, Department of Psychiatry and Mental Health, University of Cape Town.
